# Tomato Reproductive Success Is Equally Affected by Herbivores That Induce or That Suppress Defenses

**DOI:** 10.3389/fpls.2017.02128

**Published:** 2017-12-13

**Authors:** Jie Liu, Saioa Legarrea, Merijn R. Kant

**Affiliations:** Section Molecular and Chemical Ecology, Institute for Biodiversity and Ecosystem Dynamics, University of Amsterdam, Amsterdam, Netherlands

**Keywords:** mites, plant defense, induction, suppression, fruit, seed, costs, reproductive escape

## Abstract

Herbivory induces plant defenses. These responses are often costly, yet enable plants under attack to reach a higher fitness than they would have reached without these defenses. Spider mites (*Tetranychus* ssp.) are polyphagous plant-pests. While most strains of the species *Tetranychus urticae* induce defenses at the expense of their performance, the species *Tetranychus evansi* suppresses plant defenses and thereby maintains a high performance. Most data indicate that suppression is a mite-adaptive trait. Suppression is characterized by a massive down-regulation of plant gene-expression compared to plants infested with defense-inducing mites as well as compared to control plants, albeit to a lesser extent. Therefore, we hypothesized that suppression may also benefit a plant since the resources saved during down-regulation could be used to increase reproduction. To test this hypothesis, we compared fruit and viable seed production of uninfested tomato plants with that of plants infested with defense-inducing or defense-suppressing mites. Mite-infested plants produced fruits faster than control plants albeit in lower total amounts. The *T. evansi*-infested plants produced the lowest number of fruits. However, the number of viable seeds was equal across treatments at the end of the experiment. Nonetheless, at this stage control plants were still alive and productive and therefore reach a higher lifetime fitness than mite-infested plants. Our results indicate that plants have plastic control over reproduction and can speed up fruit- and seed production when conditions are unfavorable. Moreover, we showed that although suppressed plants are less productive in terms of fruit production than induced plants, their lifetime fitness was equal under laboratory conditions. However, under natural conditions the fitness of plants such as tomato will also depend on the efficiency of seed dispersal by animals. Hence, we argue that the fitness of induced plants in the field may be promoted more by their higher fruit production relative to that of their suppressed counterparts.

## Introduction

Plants have evolved multilayered defenses under pressure from pathogens and herbivores. These defenses determine the extent to which plants can escape being eaten depending on the level of susceptibility of the attacker (Schuman and Baldwin, 2016). Plant defenses may be produced constitutively or be induced specifically upon attack (Walling, 2000). Inducible defenses often are characterized by the establishment of structural reinforcements; the accumulation of toxins and inhibitors of an attacker’s digestive proteases (Kessler and Baldwin, 2002) and can include the attraction of foraging natural enemies of herbivores, e.g., via the release of distinct odors (Sabelis et al., 2001). The central regulators of these inducible defensive responses are the phytohormones jasmonic acid (JA), which orchestrates the bulk of defenses aimed at herbivores (Howe and Jander, 2008) and necrotrophic pathogens (Glazebrook, 2005), and salicylic acid (SA) which organizes defenses that primarily hamper biotrophic pathogens and phloem-feeding herbivores (Kaloshian and Walling, 2005). SA- and JA-dependent responses act often antagonistically and this may reflect an adaptive tailoring of distinct defenses against distinct attackers (Thaler et al., 2012).

It is generally assumed that defenses evolved to maximize individual inclusive fitness and that they are costly because they require resources otherwise available for growth and reproduction (Rhoades and Cates, 1976). These assumptions come together in the ‘optimal defense theory’ (Rhoades, 1979). It assumes that inducible defenses evolved not only to prevent self-intoxication but especially to minimize wasting energy on costly defenses – which may be needed only occasionally – especially in tissues that are not of vital importance for plant fitness (McKey, 1974; Coley et al., 1985; Cipollini et al., 2003). While the biological significance of costs and benefits of defenses for plant survival, growth and seed production are not always evident for every plant–herbivore system (Karban, 1993; Thaler, 1999), there are quite some studies that reported artificially manipulated defenses in various species to affect plant fitness substantially (Redman et al., 2001; Zavala et al., 2004; Accamando and Cronin, 2012). There are also data, obtained from experiments performed within a more natural ecological context, indicating that the benefits of (inducible) plant defenses outweigh their costs. For example, Baldwin (1998) treated wild populations of *Nicotiana attenuata* with the defense elicitor methyl jasmonate (MeJA) and observed that induced unattacked plants produced less seed than control unattacked plants – while for attacked plants he observed the opposite, indicating that induction can be costly yet beneficial under natural conditions. Such observations suggest that defenses often are plastic and that they are adaptive in the sense that they maximize the fitness of plants attacked by herbivores as assumed by the optimal defense theory (Schuman and Baldwin, 2016).

There are indications that plants may also have evolved regulatory traits that allow them to tailor defenses in order to minimize the ecological costs of displaying unneeded defenses that also may negatively affect beneficial organisms. Kahl et al. (2000) observed that continued attack of *N. attenuata* by the specialist herbivore *Manduca sexta* induced an ethylene-mediated response that preceded reduced nicotine accumulation in parallel with the increased emission of volatiles attractive to the caterpillar’s parasitic natural enemies. Since *M. sexta* can tolerate nicotine they reasoned that the reduction of nicotine accumulation not only prevented the plant from wasting resources but also prevented *M. sexta* larvae from sequestering nicotine as defense against the parasitoid. In addition, Voelckel et al. (2001) showed that this reduced direct defense response increased the plant’s fitness. Together this suggested that this ethylene-mediated suppression of nicotine accumulation is a plant adaptive – and not an herbivore adaptive – event.

Suppression of host defenses is a phenomenon that has been observed during diverse plant–herbivore interactions (reviewed in Kant et al., 2015) although often via experiments lacking ecological context, i.e., performed in the absence of natural competitors or enemies. In several cases suppression was shown to benefit these herbivores by improving their reproductive performance. This led to the implicit assumption that defense suppression, in most instances, will reflect an herbivore-adaptive rather than plant-adaptive trait. However, beneficial effects of defense suppression on herbivores do not necessarily exclude beneficial effects for their host plants as well possibly in the form of a plant–herbivore mutualism (Agrawal, 2000). These observations obviously warrant the question to which extent also plants may benefit from the suppression – e.g., because this could enable them to use the energy saved for increasing their reproductive output.

We addressed this question by using two species of spider mites, one that induces and one that suppresses plant defenses to infest a determinate tomato cultivar for which we could assess lifetime viable seed production. The defense-inducing spider mite *Tetranychus urticae* is a well-known pest species worldwide occurring on numerous crop plants. It harbors a large number of resistance genes that may allow it to cope with the diversity of defenses present in a diverse plant diet (Dermauw et al., 2013). Spider mites are stylet feeders (Bensoussan et al., 2016) and most species induce JA and SA defenses simultaneously on plants such as bean (Ozawa et al., 2000), tomato (Li et al., 2002; Kant et al., 2004, 2008) and Arabidopsis (Zhurov et al., 2014; Martel et al., 2015). These mites are especially sensitive to JA-mediated defenses, i.e., performance roughly doubles on JA biosynthesis or perception mutants (Li et al., 2004; Kant et al., 2008; Alba et al., 2015). To a lesser extent, they are also susceptible to SA-mediated defenses (Villarroel et al., 2016). The defense-suppressing spider mite *Tetranychus evansi* is endemic to South America but became invasive in Africa and Southern Europe during the 1970 and 1980s (Boubou et al., 2012). It suppresses both JA- and SA-mediated defenses down to – or below – housekeeping levels (Sarmento et al., 2011a) and this suppression we found to act downstream of phytohormone accumulation and to be independent of JA–SA crosstalk (Alba et al., 2015). Furthermore, *T. evansi* benefits from this suppression since it enhances its reproduction (Sarmento et al., 2011b). We could attribute suppression of defenses by spider mites to several secreted salivary effector-proteins (Jonckheere et al., 2016; Villarroel et al., 2016). *T. urticae* and *T. evansi* regularly co-occur in the field (Ferragut et al., 2013) and in laboratory experiments *T. urticae* was found to benefit from the suppression of defenses by *T. evansi* when residing in close proximity to it on the same plant by producing more offspring (Sarmento et al., 2011b). This indicates that there may be ecological costs for the defense suppressing mites associated with suppressing host defenses in natural communities due to facilitation of competitors (Glas et al., 2014; Alba et al., 2015). Moreover, suppressor-mite infested plants were still attractive to foraging predatory mites – despite the suppression of several key volatiles implicated in indirect defenses (Sarmento et al., 2011a) – and these predator species were found to predate more on spider mite prey from uninduced or suppressed plants than from induced plants, indicating yet another potential drawback of suppressing defenses within natural communities (Ataide et al., 2016). Taken together, while the direct fitness benefits of defense suppression for mites that monopolize their feeding site are obvious, it is less clear if and how they prevent this from backfiring when facilitating their competitors and natural enemies under natural conditions. Possibly the production of large amounts of web to exclude predators and competitors (Sarmento et al., 2011b), local hyper-suppression of defenses in response to defense-inducing invaders (Schimmel et al., 2017a,b) as well as reproductive interference of *T. urticae* females by *T. evansi* males (Sato et al., 2014) counteract some of these apparent ecological costs.

Until now, our research has focused primarily on the mite side of this interaction. However, the question to which extent plants allow their defenses to be suppressed – possibly as a tolerance strategy – remains unanswered (Agrawal, 2000). The mere existence of a rich diversity of defenses across the plant kingdom suggests that defense suppression will on average be more detrimental to plant fitness than induction will be, especially when imposed by herbivores that overexploit their host rapidly, like spider mites. However, it was also found that plants under stress have a certain degree of plastic control over their reproduction and may display reproductive escape during the early phase of the infestation by producing fruits and seeds earlier in their lifecycle phenology than when not attacked (Lucas-Barbosa et al., 2013). If so, we reasoned, plants may actually benefit from massive downregulation of genes and enzymes involved in defenses compared to induced plants and even to control plants albeit to a lesser extent (Kant et al., 2008; Sarmento et al., 2011a; Alba et al., 2015; Godinho et al., 2016; Schimmel et al., 2017a) because the resources saved could be used to increase reproduction. Hence, we assessed lifetime reproductive fitness – i.e., the number of viable seeds produced per plant (Semel et al., 2006) – of the determinate tomato variety Micro-Tom (Marti et al., 2006) when infested with the defense inducer *T. urticae* or infested with the defense suppressor *T. evansi* under controlled conditions.

## Materials and Methods

### Plant and Herbivore Rearing

Tomato determinate cultivar Micro-Tom (*Solanum lycopersicum* L.) plants were used in the experiments. Micro-Tom is a widely used model plant for research on tomato genomics and physiology, including the development and metabolism of fruit (Matsukura et al., 2008; Campos et al., 2010). One of the main advantages of this cultivar is its relatively small size and its short determinate life cycle (Meissner et al., 1997). Micro-Tom seedlings were grown in a greenhouse with day/night temperatures of 23–18°C and a 16/8 h light/dark regime. Three days prior to each experiment all plants were transferred to a climate room at 23–18°C, a 16/8 h light regime with 300 μEm^-2^ s^-1^, and 60% relative humidity (RH).

For an herbivore that induces plant defenses we used the two-spotted spider mite line *T. urticae* Koch strain Santpoort-2, which induces a strong JA- and SA-response (Kant et al., 2008). Mite colonies of *T. urticae* Santpoort-2 were maintained on detached leaves of *Phaseolus vulgaris* L. cv. Speedy. For an herbivore that suppresses plant defenses we used the red spider mite line *T. evansi* Baker & Pritchard strain Viçosa-1, which suppresses JA- and SA-responses (Kant et al., 2008). Mite colonies of *T. evansi* Viçosa-1 were maintained on detached leaves of *S. lycopersicum* cv. Castlemart. The cultures were maintained in a climate room at 23–18°C, a 16/8 h light regime with 300 μE m^-2^ s^-1^, and 60% RH. For the experiments, so-called egg waves were produced to obtain mites of the same age + 1 day (Kant et al., 2008) on tomato cv. Castlemart and from these we used 2-day-old adult female mites for infesting the Micro-Tom plants.

### Plant Infestation with Spider Mites

Micro-Tom plants 24 days after sowing were infested with 2-day-old adult female *T. urticae* Santpoort-2 or *T. evansi* Viçosa-1 and uninfested plants were used as control. For each treatment we used five plants. For each plant, five adult females were placed on the adaxial surface of the oldest fully expanded leaflet using a soft paintbrush. Uninfested plants were also touched in a similar way with a paintbrush. Plants were grown in pots (0.74 L) with soil and these were separated by a water barrier. The pots contained commercial potting soil (Soil Nr. 3, Jongkind Grond B.V., Aalsmeer, The Netherlands) and we did not add extra fertilizer during the course of the experiment. The plants were watered once per week by adding water to the saucer. These conditions are identical to those we used previously, e.g., in Kant et al. (2004, 2008) and Alba et al. (2015). We took photos from the same position from every plant weekly to record their phenotypes. This experiment was conducted three times independently over a year.

### Tomato Defense Gene Expression

We wanted to confirm induction and suppression of defenses by our mite lines in Micro-Tom as observed in other tomato varieties. To do so we used the well-established defense marker genes *PI-IIc* (also called WIPI-II, Farmer et al., 1992) and *PR-1a* (Van Loon and Van Strien, 1999) which mark mite-activated jasmonate and salicylate defenses respectively in several indeterminate tomato varieties (Kant et al., 2008; Sarmento et al., 2011a; Alba et al., 2015). Micro-Tom tomato plants of 21 days old were infested with 15 adult female spider mites per leaflet using three leaflets per plant. We used five plants per treatment. Seven days after infestation, when the difference in magnitude between induction and suppression of the *PI-IIc* and *PR-1a* defense genes are maximal (Alba et al., 2015), leaflets were harvested. Gene expression was assessed by means of qRT-PCR as described by – and using the same primers as – Alba et al. (2015). Transcript abundances were normalized to actin and for plotting the data we scaled to the lowest mean value. Prior to statistical evaluation, data were log-transformed if necessary to meet the assumption of homogeneity of variance as assessed by Levene’s test. Differences among treatments were assessed with ANOVA with ‘treatment’ as factor. Means of each group were compared by LSD *post hoc* tests in IBM SPSS Statistics 22.

### Fruit Production

Plants were inspected weekly. Green and red fruits were counted separately until the end of experiment. The endpoint of the experiment was chosen arbitrarily as the moment when mite-infested plants had not produced a new green fruit for the third week in a row. Total number of fruits per plant at the endpoint of the experiment were analyzed using ANOVA with ‘treatment’ and ‘independent repetition’ as factors (three treatments; five plants per treatment per repetition, three independent repetitions in time). Means of each group were compared by LSD *post hoc* test using IBM SPSS Statistics 22.

The total number of fruits and the number of red fruits per plant over the time course of the experiment were plotted and fitted with regression lines using a sigmoidal Chapman-Richards model. The repeated measurements of the total number of fruits and red fruits number per plant across time were analyzed with a linear mixed effects model (LME) (Pinheiro et al., 2014) in R 3.3.1 (package nlme), with ‘number of fruits’ as the dependent variable, with ‘treatment’ and ‘time’ (weeks) as fixed factors and ‘plant’ as a random factor. Finally, differences between treatments per time point (week) were tested using a LME followed by general linear hypothesis testing (glht) to analyze the contrasts via Tukey using the Least-squares means (lsmeans) package in R (Lenth, 2016).

### Viable Seeds Produced by Infested and Uninfested Plants

All the tomato fruits were harvested separately per plant at the end of the experiment. Fruits were numbered, washed and seeds were collected and dried on tissue paper. Only the seeds from the ripe fruits were used for the following experiments (note that only some of the control plants still contained occasional green fruits at the end of the experiment). We pooled the seeds per treatment-group per independent repetition and randomly selected 50 seeds per group. These seeds were allowed to germinate on moist filter paper being the first 7 days in the dark. The percentage of viable seeds (i.e., the seeds that germinated) was then calculated per group of 50 seeds. This percentage was then used to predict the total number of viable seeds per plant for that particular treatment group. For each of the three repetitions of this experiment this calculation was performed separately. Analysis of variance (ANOVA) with ‘treatment’ and ‘independent repetition’ as factors and least significant difference (LSD) *post hoc* test were used to compare means of each group using IBM SPSS Statistics 22.

### Root, Shoot, and Fruit Biomass

These following measurements were performed only on the plants of the third replicate experiment (*n* = 5 for each treatment). Every fruit that was harvested was weighed on a microbalance before the seeds were harvested. Also all of the remaining plant shoot tissue was harvested and weighed on a microbalance under the assumption that the mass of the mites and their webbing were neglectable, i.e., a single adult female weighs roughly 200 ng (Walling et al., 1968). Roots were harvested from the soil, cleaned in water, gently dried with tissue paper and also weighed on a microbalance. We subsequently calculated total plant fresh weight (shoots plus roots) across the treatments (Supplementary Figure 2A); fruit fresh weight relative to plant fresh weight (shoots plus roots) across the treatments (Supplementary Figure 2B) and the number of viable seeds relative to total mass (shoots plus roots plus fruits) (Supplementary Figure 2C). Results were evaluated using ANOVA followed by LSD *post hoc* test to compare means of each group using IBM SPSS Statistics 22.

## Results

### Tomato Defense-Gene Expression

**FIGURE 1:**
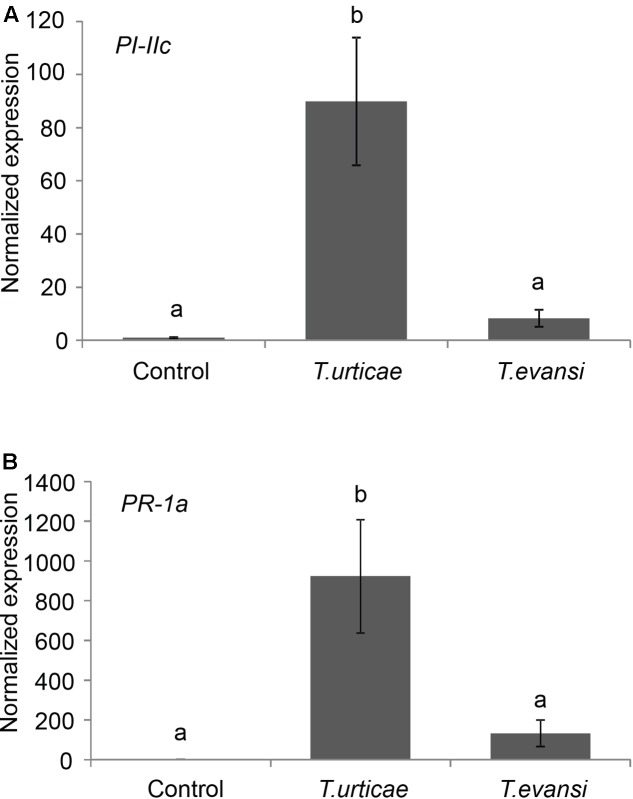
Relative transcript abundances of defense-related genes in *Tetranychus evansi* and *Tetranychus urticae* infested Micro-Tom leaflets at 7 days post-infestation. *PI-IIc* **(A)** and *PR-1a* **(B)** transcript levels were relative to actin. Uninfested leaflets were used as controls. The bars represent the means ( ± SE) and are scaled to the lowest mean by putting this to 1. Bars annotated with different letters were significantly different according to Fisher’s LSD test (*P* < 0.05) after ANOVA, *n* = 5.

Gene expression data confirm that *T. urticae* Santpoort-2 induces defense gene expression whereas *T. evansi* does not in Micro-Tom. *T. urticae* Santpoort-2 significantly upregulated the *PI-IIc* and *PR-1a* expression in Micro-Tom compared to uninfected control leaflets at 7 dpi (**Figure 1**; *F*_2,11_ = 18.56, *P* < 0.001; *F*_2,12_ = 14.56, *P* < 0.001), In contrast, *T. evansi* did not upregulate the expression of PI-IIc or PR-1a above control levels significantly in Micro-Tom (**Figure 1**; *F*_2,11_ = 18.56, *P* = 0.68; *F*_2,12_ = 14.56, *P* = 0.49).

### Fruit Production during the Course of the Infestation

**FIGURE 2:**
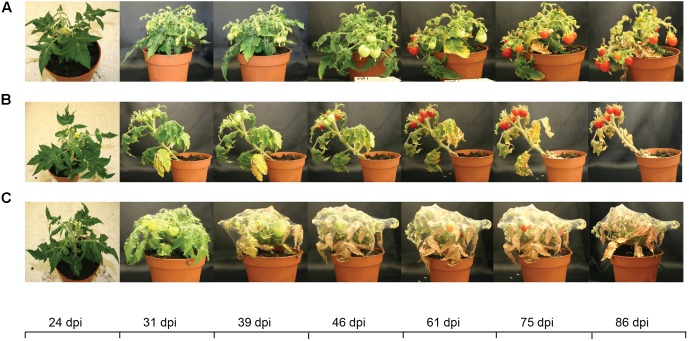
Micro-Tom tomato infested with spider mites over the course of the experiment [days post-infestation (dpi)]. **(A)** Uninfested control tomato plants. **(B)** *T. urticae*-infested tomato plants. **(C)** *T. evansi*-infested tomato plants.

The effect of the infestation became clearly visible around 4 weeks after infestation because around this time point the mite population started to migrate across the plant (**Figure 2**). At earlier time points the infestation was still largely restricted to the infested leaflets [see 24 days post-infestation (dpi) in **Figure 2**]. Around 4 weeks after infestation the first green fruits appeared in the mite-infested plants. Around 4–5 weeks after infestation the extreme web production by *T. evansi* (Sarmento et al., 2011b) became clearly visible as it covered a substantial part of the plant’s canopy. After around 6–7 weeks the first ripe fruits appeared in the mite-infested treatments. When plants had not produced a new green fruit for 3 weeks in a row we stopped the experiment (about 86 dpi). At this point practically all fruits were fully ripe although in some of the control plants there were still some unripe fruits left (39 in total across the three independent repetitions).

**FIGURE 3:**
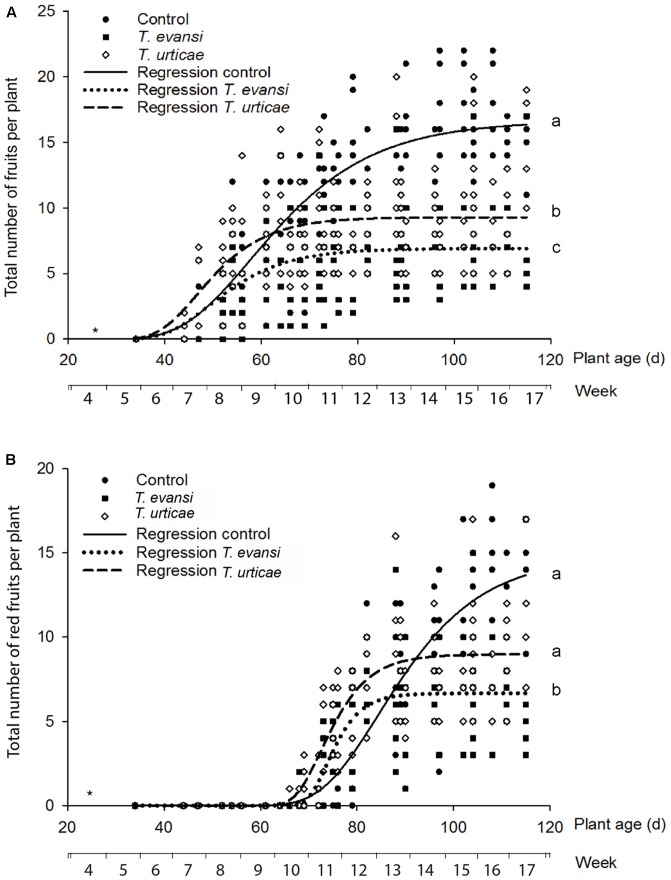
Total fruits **(A)** and red fruits **(B)** production per plant of spider mites infested Micro-Tom plants in time series post-infestation. The data were fitted to the regression equation: f = a^∗^(1-exp(-b^∗^x))ˆc. The solid line shows the average total number of fruits for uninfested control plants (closed circles) (**A**: *R*^2^ = 0.79, *P* < 0.0001; **B**: *R*^2^ = 0.86, *P* < 0.0001). The dotted line shows the average total number of fruits for the *T. evansi* infested plants (closed rectangles) (**A**: *R*^2^ = 0.39, *P* < 0.0001; **B**: *R*^2^ = 0.67, *P* < 0.0001). The dashed line shows the average total number of fruits for *T. urticae* infested plants (open circles) (**A**: *R*^2^ = 0.45, *P* < 0.0001; **B**: *R*^2^ = 0.75, *P* < 0.0001). Differences in mean fruit production per plant among treatments over time were tested by means of a linear mixed effects (LME) model using the number of fruits and plant as a random factor. Different letters next to the curves indicated overall significant differences among treatments (lsmeans of R, *P* < 0.05). Asterisk (^∗^) indicated the age of plants when infested with mites.

We first analyzed the total number of fruits per treatment at the end of the experiment (i.e., after mite-infested plants had stopped producing new fruits for over 3 weeks). There was a significant difference in the number of total fruits produced over the course of the experiment across all the three treatments and the interaction between treatment and time was significant (**Figure 3A**; LME: Chi^2^ = 85.91, df = 2, *P* < 0.0001). To analyze the differences between treatments per time point, ‘plant age’ was converted to a categorical scale (‘weeks,’ i.e., 1–7 days after sowing = week 1, 8–14 days after sowing = week 2, etc.) and thus as categorical factor. No statistically significant differences were found between the treatments before week 10. At week 11, the total number of fruits of the *T. evansi* and *T. urticae* infested plants and the uninfested control plants differed significantly (**Figure 3A**; glht: control vs. *T. urticae*: *P* = 0.048; control vs. *T. evansi*: *P* = 0.001). Then from week 13 onward the total number of fruits produced by uninfested control plants was higher than those of infested plants (**Figure 3A**; glht: week 13: control vs. *T. urticae*: *P* = 0.003; control vs. *T. evansi*: *P* < 0.001; week 14: control vs. *T. urticae*: *P* < 0.001; control vs. *T. evansi*: *P* < 0.001). From week 15 onward, the difference in the total number of fruits among all three treatments was statistically significant (**Figure 3A**; glht: week 15: control vs. *T. urticae*: *P* < 0.001; control vs. *T. evansi*: *P* < 0.001; *T. urticae* vs. *T. evansi: P* = 0.04; week 16: control vs. *T. urticae*: *P* < 0.001; control vs. *T. evansi*: *P* < 0.001; *T. urticae* vs. *T. evansi: P* = 0.03). Finally, the regression lines in **Figure 3A** had a significant fit for each of the treatments: for plants infested with *T. urticae R*^2^ = 0.45 and *P* < 0.0001; for plants infested with *T. evansi* the *R*^2^ = 0.39 and *P* < 0.0001 and for the uninfested control plants the *R*^2^ = 0.79 and the *P* < 0.0001 where the *P*-values indicate the difference of the fitted line from slope = zero.

We subsequently analyzed the total number of ripe fruits per treatment at the end of the experiment. There was a significant difference in the number of ripe fruits produced over the course of the experiment across all the three treatments and the interaction between treatment and time was significant (**Figure 3B**; Chi^2^ = 33.16, df = 2, *P* < 0.0001) indicating that the timing of fruit ripening differed across treatments. Until week 10 there were no significant differences among the treatments, but at week 11 *T. urticae*-infested plants carried more ripe fruits than uninfested control plants (**Figure 3B**; glht: *P* = 0.03). From week 13 onward, the number of ripe fruits did not increase anymore on the infested plants while uninfested control plants continued to produce red fruits. At week 14 there was a significant difference in the number of total red fruits between *T. evansi* infested plants and uninfested control plants (**Figure 3B**; glht: *P* = 0.02). From week 15 to 16, there was a significant difference in the number of total fruits among all three treatments (**Figure 3B**; glht: week 15: control vs. *T. urticae*: *P* = 0.03; control vs. *T. evansi*: *P* < 0.001; *T. urticae* vs. *T. evansi: P* = 0.02; week 16: control vs. *T. urticae*: *P* < 0.001; control vs. *T. evansi*: *P* < 0.001; *T. urticae* vs. *T. evansi: P* = 0.02). Finally, the regression lines in **Figure 3B** had a significant fit for each of the treatments: plants infested with *T. urticae* had an *R*^2^ = 0.75 with a *P* < 0.0001; plants infested with *T. evansi* had an *R*^2^ = 0.67 with a *P* < 0.0001) and uninfested control plants had an *R*^2^ = 0.86 with a *P* < 0.0001.

**FIGURE 4:**
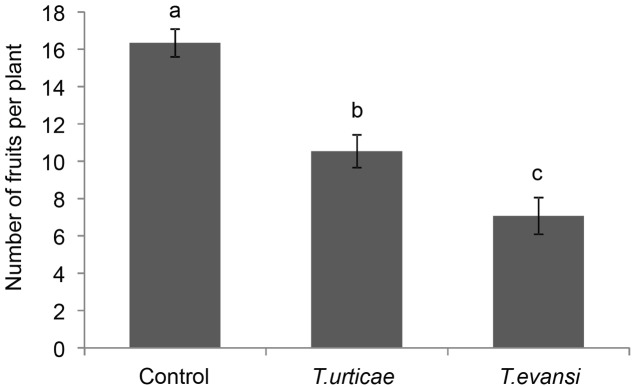
Average total number of fruits per plant (±SE) of uninfested Micro-Tom plants in comparison to plants infested with *T. urticae* or *T. evansi*. Bars annotated with different letters were significantly different according to Fisher’s LSD test (*P* < 0.05) after ANOVA with *n* = 15 per treatment.

At the end of the experiment, the total number of fruits produced was significantly different among treatments (**Figure 4**; *F*_2,42_ = 25.77, *P* < 0.001). The total number of fruits produced by infested plants was significantly lower than for uninfested plants (**Figure 4**; *P* < 0.001), and *T. urticae*-infested plants had produced significantly more fruits than *T. evansi*-infested plants (**Figure 4**; *P* = 0.011).

### Viable Seeds Produced by Infested and Uninfested Plants

**FIGURE 5:**
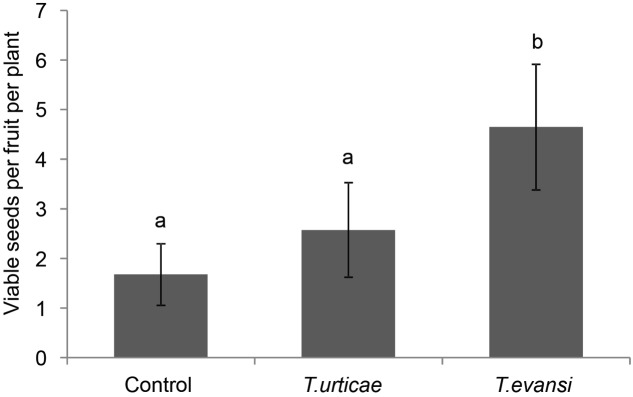
Average viable seeds per fruit per plant (±SE) of uninfested Micro-Tom plants in comparison to plants infested with *T. urticae* or *T. evansi*. Bars annotated with different letters were significantly different according to Fisher’s LSD test (*P* < 0.05) after ANOVA with *n* = 15 per treatment.

**FIGURE 6:**
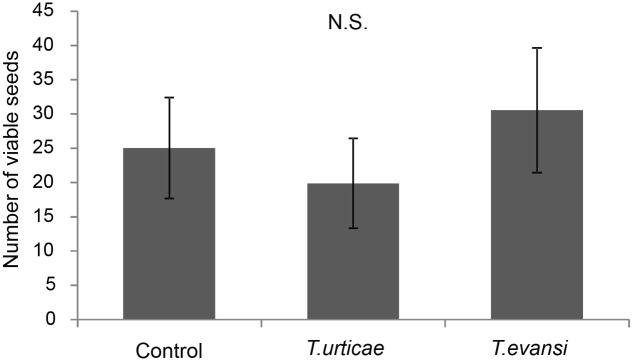
Reproductive fitness (average viable seeds per plant ±SE) of Micro-Tom plants infested with spider mites. Bars indicate the average number of viable seeds per plant of uninfested Micro-Tom plants (control) or plants infested with spider mites (*T. urticae*, *T. evansi*). Seeds were considered viable when they germinated. N.S. indicates that no significant differences among treatments were detected using ANOVA (*n* = 15 plants per treatment).

While *T. evansi*-infested plants produced the lowest number of fruits, these fruits contained significantly more seeds than the fruits obtained from the other treatments (1.68 ± 0.62) (mean ± SE) in the control; 2.547 ± 0.95 in the fruits of *T. urticae*-infested plants and 4.65 ± 1.27 for the fruits from *T. evansi*-infested plants) (**Figure 5**; *F*_2,42_ = 3.27, *P* = 0.048). Therefore their total seed production did not differ (*F*_2,42_ = 0.45, *P* = 0.64). Also when correcting for the percentage of germination (Supplementary Figure 1), i.e., when determining the number of viable seeds, the control plants and the *T. urticae*- or *T. evansi*-infested plants were not statistically different (**Figure 6**; *F*_2,42_ = 0.21, *P* = 0.81). Finally, when inferring the extra number of viable seeds from the green fruits that had remained on some of the control plants at the end of the experiment, we did not detect significant differences among treatments (data not shown; *P* > 0.05). Thus the plants had produced equal numbers of viable seeds.

### Root, Shoot, and Fruit Biomass

Total plant fresh weight (shoots plus roots) was lower for the *T. evansi* infested plants than for the other two treatments (Supplementary Figure 2A) (*F*_2,12_ = 7.89, *P* = 0.007) predominantly due to a lower shoot biomass. The average weight of the fresh fruits relative to the total plant biomass did not differ across treatments (Supplementary Figure 2B) (*F*_2,12_ = 0.58, *P* = 0.58) although there was a trend toward relatively larger fruits in the mite-infested plants. Finally also the number of viable seeds relative to total mass (shoots plus roots plus fruits) did not significantly differ (Supplementary Figure 2C) (*F*_2,12_ = 0.66, *P* = 0.54) although the number of seeds for the *T. urticae*-infested plants varied considerably in this third replicate experiment.

## Discussion

We tested to which extent induction and suppression of defenses by herbivores affect the fitness of determinate tomato plants differentially. We observed that mite-infested plants produced fruits faster than control plants albeit in lower total amounts. We also observed that the *T. evansi*-infested plants – of which the defenses were suppressed – produced the lowest number of total (ripe) fruits. However, the number of viable seeds was equal across treatments at the end of the experiment irrespective of induction or suppression. Therefore we argue that there are no indications that defense suppression by *T. evansi* allows a plant to reallocate extra resources to reproduction compared to plants infested with defense inducing *T. urticae*.

### Defense-Suppression by Mites Does Not Reflect a Tomato-Adaptive Trait

Micro-Tom is a cultivated tomato and hence the magnitude and timing of its responses may not be directly comparable to that of wild relatives (Bleeker et al., 2012; Escobar-Bravo et al., 2016; Whitehead et al., 2016). However, there are no obvious arguments why the comparison between a cultivated plant infested with different mite species could not serve as a model for plants such as tomatoes in general since both wild and cultivated plants display induced defenses (Haak et al., 2014; Turcotte et al., 2014). Induced defenses are widespread and are believed to represent a form of adaptive phenotypic plasticity (Agrawal, 2001) that enable plants to cope with pests such as herbivores (Gotthard and Nylin, 1995; DeWitt et al., 1998; Agrawal, 1999a,b). Inducibility may augment constitutive display of defenses by minimizing the overall costs (Cipollini et al., 2003) and by limiting the negative effects of auto-toxicity (Baldwin and Callahan, 1993; Nabity et al., 2009). Plant defenses may impose two types of costs: (a) physiological costs such as energetic costs related to the use of resources that otherwise could be used to increase fitness (Agrawal et al., 1999) and (b) ecological costs for example by excluding natural enemies of herbivores (Eisner et al., 1998) or by promoting other types of attackers as a consequence of regulatory trade-offs (Thaler et al., 1999). For this study we were interested in the physiological costs of induced defenses. Inducing defenses artificially in solanaceous plants was shown previously to reduce seed production (as a proxy for fitness). For example, Baldwin (1998) treated wild tobacco *N. attenuata* with MeJA in the field and observed reduced seed production in treated unattacked plants. Similarly, Redman et al. (2001) treated an indeterminate tomato variety in the greenhouse with a JA-containing solution and observed a delay and a reduction in total fruit set and an overall lower production of seeds. However, such costs were not always observed since Thaler (1999) found that JA-induced field-grown tomato plants, although producing fewer flowers than control plants, did not produce fewer fruits. Similarly, our data suggest that lifetime (viable) seed production actually is alike between herbivore-infested and control plants. Also when calculating the predicted viable seed production including the expected number of seeds from the green fruits left on control plants at the end of the experiment, we did not detect significant differences. However, tomatoes are often perennial and uninfested varieties with a longer lifespan than our determinate variety would likely have produced more seeds during their indeterminate lifetime when uninfested than when overexploited by mites and thus reach a higher reproductive fitness. Moreover, the fact that our control plants exhibited the same lifetime reproductive fitness as the mite-infested plants emphasizes that the latter do not overcompensate their reproductive fitness as was sometimes observed in mechanically damaged Arabidopsis (Scholes et al., 2016) and tomatoes damaged by omnivores (Sanchez and Lacasa, 2008). Possibly, suppression of defenses does leave a plant with more resources available for producing seeds but this advantage may be counter-acted by the increased feeding activities of the suppressor mite since these have a higher peak reproductive output (Alba et al., 2015). *T. urticae* and *T. evansi* overexploit tomato plants and in previous experiments working under similar conditions we showed the moment of overexploitation, i.e., after which the plant has been consumed and the mite population collapses, for *T. urticae* to occur roughly a week later than for *T. evansi* (Sarmento et al., 2011a). Hence, when correcting for mite densities the fruit set curves (**Figure 3A**) may shift around one week closer together. However, this has no consequences for the lifetime fruit production (**Figure 4**). While seed production did not differ in our experiments we did observe a reduction in fruit production. This may point to an ecological cost of defense suppression by herbivores as fewer fruits might decrease the chances of seed dispersal by fruits-eating animals (Moyle, 2008). Taken together, our data suggest that the costs for dealing with defense suppression by herbivores will be ecological rather than energetic and hence we did not find indications for it reflecting a plant-adaptive response.

### Herbivore-Induced Plasticity in Fruit Production

Local biotic interactions with pollinators and herbivores are believed to shape local flower phenology through natural selection (Elzinga et al., 2007; Kessler et al., 2010). However, our data show that such responses can be plastic as well. How the plant’s phytohormones orchestrate such plastic responses is poorly understood. Fruit set was shown to involve a complex temporal interplay between auxins, cytokinins, brasinosteroids, and gibberellins and followed by ethylene and ABA during ripening (McAtee et al., 2013). In addition, also the genetic basis of plant growth and development is currently still largely descriptive (Gapper et al., 2014) but elucidating these processes in the future will give breeders access to custom tools for optimizing plant productivity. We observed that tomato plants infested with mites flower earlier and produce (ripe) fruits earlier than control plants. This shows that the timing of tomato fruit production is not hard-wired to the plant’s life cycle. For diverse single and multi-cellular organisms it was shown that increased levels of stress can result in increased allocation to reproduction, including *Trifolium repens* upon attack by herbivorous snails (Griffiths and Bonser, 2013). Moreover, Lucas-Barbosa et al. (2013) observed that annual plant *Brassica nigra* responded to *Pieris brassicae* eggs by accelerating seed production and thereby prevented flower herbivory. They referred to this phenomenon as ‘reproductive escape’ and classified it as a defense. In fact, *T. evansi*-infested plants produced not only less fruits, but also their biomass was lower at the end of the experiment (note: this was only assessed for the third replicate). Fruit biomass as a proportion of total biomass at the end of the experiment was similar across the treatments (between 12 and 17%; Supplementary Figure 2B) indicating that the relative investment in fruit production is largely unaffected by mite infestation. However, even though suppressed plants have overall less energy or resources to invest in reproduction they upheld their reproductive fitness by producing more viable seeds per fruit. The reproductive-escape response of the tomatoes in our study clearly was very efficient for dodging the destructive effects of a large spider mite population. However, while Micro-tom tomato is a self-pollinating variety (Marti et al., 2006) many of the wild varieties in the field depend on cross pollination (Chetelat et al., 2009). Hence, for wild tomatoes flowering early could negatively affect fruit and seed production, e.g., when the availability of natural pollinators is lower earlier in the season (Kessler et al., 2010). In addition, plants with earlier fruit production and ripening might risk losing fruits due to early periods of frost and other environmental disadvantages and may increase the chance of being eaten by seed predators (Johnson et al., 2015). We observed that tomato plants infested with spider mites produced fruits not only earlier but also produced them in lower numbers (albeit containing more seeds) and of these the plants infested with defense-suppressing mites produced the lowest numbers. Plant reproductive fitness will depend not only on the number of viable seeds per plant but also on the success of these seeds to germinate, survive, and reproduce. An important factor that influences this success is seed dispersal. The efficiency of seed dispersal by animals depends on several factors, including the number of seeds dispersed per visit (Schupp, 1993), and the more seeds are dispersed by less individual dispersers the higher the risk these seeds end up in the same area and thus will have to compete with each other (Campbell et al., 2017). In addition, not only reduced but also mistimed fruit production may affect inclusive fitness negatively, i.e., when there is a mismatch between the moment of ripening and the presence of seed dispersers (Howe and Miriti, 2004) such as those of wild tomato seeds (Pearce et al., 1988; Caceres and Moura, 2003; Moyle, 2008). Thus while the reproductive success of the tomatoes infested by mites may be maintained via a reproductive escape response, their reproductive fitness in the field may well be lower. Therefore we argue that under natural circumstances plants infested with suppressor mites, on average, will probably end up with the lowest inclusive fitness, since for these plants the distribution of seeds across fruits is the most unfavorable. Finally one may wonder if cultivated tomato is a good model for wild plants. Cultivated and wild plants may differ considerably (VanDoorn and de Vos, 2013; Whitehead et al., 2017). However, cultivated tomatoes treated with JA produce fewer fruits and fewer seeds per plant (Redman et al., 2001) like also wild solanaceous plants do (Baldwin, 1998). Hence for assessing the relative costs of defenses induced by different attackers, cultivated tomato seems to be a suitable model.

Collectively our data indicate that defense suppression by *T. evansi* does not improve the reproductive output of tomato plants but also that infestation not necessarily reduces it. Our data show that plants can have sufficient plastic control over the timing of flowering and fruit production to display an efficient reproductive escape response when infested with mites that either induce or that suppress plant defenses. These effects were not caused by differences in population growth between the two mite species because, if so, plants infested with *T. evansi* should have produced the lowest number of seeds. However, if the efficiency of this reproductive escape across treatments would uphold under natural conditions is doubtful since earlier flowering may interrupt interactions with pollinators and reduced fruit production – despite the equal total seed production among the treatments – may interfere with optimal seed dispersal. If so, this would reinforce the notion that defense suppression is not a plant-adaptive trait. Ideally our experiments would have been performed with JA-mutants – such as *def-1* or *coi-1* (Li et al., 2001) – as well since that would have allowed us to discriminate between the true energetic costs of displaying defenses and the effects due to differences in mite-population growth. However, technically this is not feasible since JA-mutants are pleiotropic and severely impaired in seed production. Hence we argue that despite their physiological plasticity, the ecological costs for plants challenged by defense-suppressing herbivores in nature may be larger than for plants infested with defense-inducing mites and that this will result in suppressed plants having the lowest fitness. This would indicate that ecological rather than physiological costs are responsible for positive selection of plant defenses in tomato.

## Author Contributions

MK originally formulated the idea. JL, SL, and MK conceived and designed the experiments. JL and SL performed the experiments. JL and SL analyzed the data. JL, SL, and MK wrote the manuscript.

## Conflict of Interest Statement

The authors declare that the research was conducted in the absence of any commercial or financial relationships that could be construed as a potential conflict of interest.
