# Supplementary material for: Tomato Reproductive Success Is Equally Affected by Herbivores That Induce or That Suppress Defenses
Source: Front Plant Sci. 2017 Dec 13;8:2128. doi: 10.3389/fpls.2017.02128 (PMC5733352; doi:10.3389/fpls.2017.02128)
Supplement: Supplementary file 1 [file Image_1.PDF]

Supplementary Material

Tomato reproductive success is equally affected by herbivores that induce or that suppress defenses

Jie Liu, Saioa Legarrea, Merijn R. Kant*

*** Correspondence:** Corresponding Author: m.kant@uva.nl

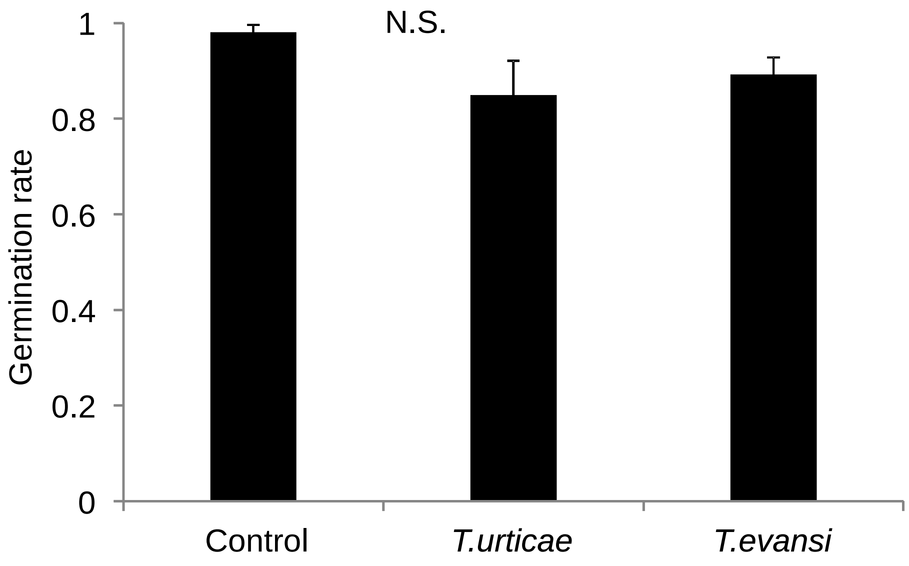

Supplementary Figure 1. Seeds germination rates. The figure shows the germination rate of the seeds across the treatments. Bars annotated with different letters were significantly different according to Fisher's LSD test ($P < 0.05$) after ANOVA.

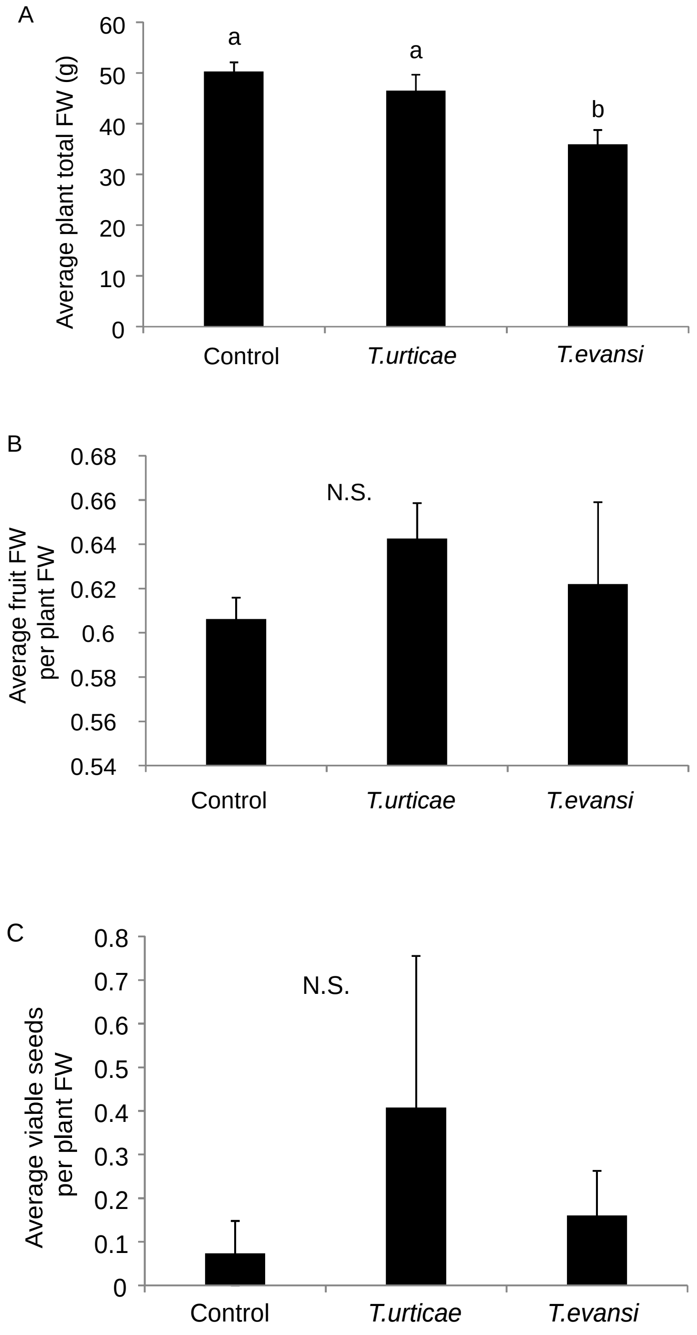

Supplementary Figure 2. The effect of the treatments on plant biomass as obtained from the third replicate experiment. The figure shows (**A**) total plant fresh weight (shoots plus roots) across the treatments, (**B**) fruit fresh weight relative to plant fresh weight (shoots plus roots) across the treatments and (**C**) viable seeds relative to total mass (shoots plus roots plus fruits). FW = fresh weight.
